# Role of Toll-Like Receptors in Immune Activation and Tolerance in the Liver

**DOI:** 10.3389/fimmu.2014.00221

**Published:** 2014-05-16

**Authors:** Nobuhiro Nakamoto, Takanori Kanai

**Affiliations:** ^1^Division of Gastroenterology and Hepatology, Department of Internal Medicine, Keio University School of Medicine, Tokyo, Japan

**Keywords:** Toll-like receptor, Kupffer cell, dendritic cell, liver tolerance, microbiota

## Abstract

Liver has a unique vascular system receiving the majority of the blood supply from the gastrointestinal tract through the portal vein and faces continuous exposure to foreign pathogens and commensal bacterial products. These gut-derived antigens stimulate liver cells and result in a distinctive immune response via a family of pattern recognition receptors, the Toll-like receptors (TLRs). TLRs are expressed on Kupffer cells, dendritic cells, hepatic stellate cells, endothelial cells, and hepatocytes in the liver. The crosstalk between gut-derived antigens and TLRs on immune cells trigger a distinctive set of mechanisms to induce immunity, contributing to various acute and chronic liver diseases including liver cirrhosis and hepatocellular carcinoma. Accumulating evidence has shown that TLRs stimulation by foreign antigens induces the production of immunoactivating and immunoregulatory cytokines. Furthermore, the immunoregulatory arm of TLR stimulation can also control excessive tissue damage. With this knowledge at hand, it is important to clarify the dual role of disease-specific TLRs as activators and regulators, especially in the liver. We will review the current understanding of TLR signaling and subsequent immune activation and tolerance by the innate immune system in the liver.

## Introduction

**Figure 1:**
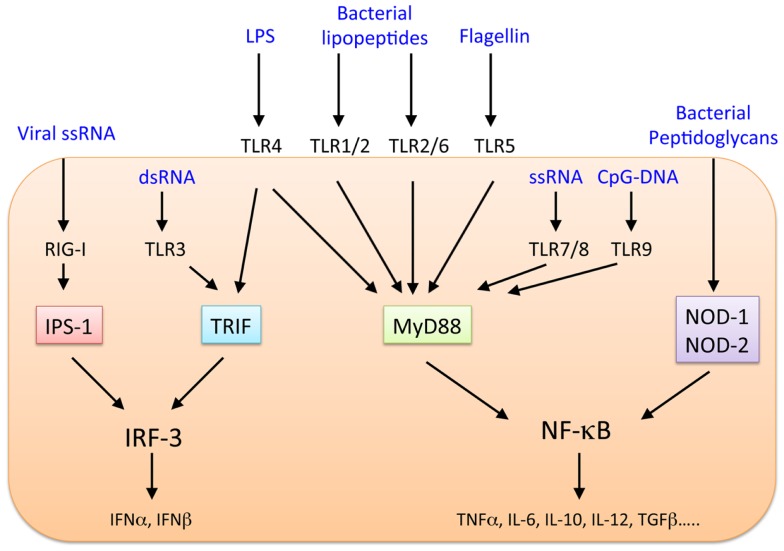
**TLRs and downstream signaling pathways**.

The liver faces continuous exposure to many pathogens and commensal bacterial products, and the innate and adaptive immune responses of the liver favor the induction of immunological activation and tolerance as appropriate (1–5). Although various immune compartments, such as T cells including CD4^+^CD25^+^Foxp3^+^ regulatory T cells (Tregs), natural killer (NK) cells, natural killer T (NKT) cells, macrophages [Kupffer cells (KC)], conventional or classical dendritic cells (cDCs), and plasmacytoid DCs (pDCs), reside in the normal liver (1, 2), it is unknown which types of cells induce inflammation and tolerance and how these cells work together to maintain immunological balance. The innate immune system is thought to play a major role in maintaining homeostasis in the liver. Gut-derived bacterial products enter the liver through the portal vein. However, liver inflammation usually does not occur because the intact mucosal barrier system of the healthy intestine prevents translocation of microbial products. When this barrier is disrupted, bacteria translocate to the liver in large quantities, resulting in the activation of the hepatic innate immune system. Cells within the hepatic sinusoids express a receptor that recognizes lipopolysaccharide endotoxin (LPS), expressed in the outer membrane of Gram-negative bacteria, and effectively remove this molecule. The Toll-like receptors (TLRs) recognize pathogen-associated molecular patterns (PAMPs) as part of innate immune defenses against foreign pathogens, including bacteria, DNA and RNA viruses, fungi, and protozoa (6, 7). Thirteen mammalian TLRs have been identified, and TLR1–10 are expressed in humans. TLRs, their ligands, and downstream signaling pathways are shown in Figure 1. In general, the healthy liver contains low mRNA levels of TLRs and their downstream signaling molecules, such as myeloid differentiation primary response gene-88 (MyD88), compared with other organs (8, 9). The continuous antigen exposure and recognition via TLRs in the liver may trigger a distinctive set of mechanisms to maintain self-tolerance and induce immunity against infection depending on the particular situation. Here, we will review the dual role of TLRs as activators and regulators of immune responses in the liver.

## TLR Signaling in the Liver

In the liver, hepatocytes account for 60–80% of the total cell population (10). Non-parenchymal cells consist of KCs, DCs, lymphocytes, hepatic stellate cells (HSCs), liver sinusoid endothelial cells (LSECs), and biliary cells. Each cell population exhibits a different TLR expression.

### Kupffer cells

Kupffer cells are hepatic-resident macrophages and account for about 20% of the non-parenchymal cells in the liver. KCs engage in phagocytosis and antigen presentation, and they are the primary cells that encounter gut-derived toxins such as LPS and orchestrate immune responses within the liver (11). Accordingly, KCs express TLR4 and are responsive to LPS (12). KCs also express TLR2, TLR3, and TLR9 and respond to their ligands (13–15). Following LPS stimulation, KCs produce tumor necrosis factor α (TNFα), interleukin (IL)-1β, IL-6, IL-12, and IL-18 (16). However, KCs also release anti-inflammatory cytokines such as IL-10 in response to continuous stimulation with low levels of LPS, a phenomenon known as LPS tolerance (17). Similarly, several murine experiments demonstrated a role for macrophages in restricting inflammatory responses during the recovery phase of liver injury (18). These results indicate that KCs act as both immune activating and immune regulatory cells depending on the specific situation.

### Dendritic cells

Hepatic DCs account for a very small proportion (<1%) of non-parenchymal cells in the liver. DCs in lymphoid and non-lymphoid tissues are classified into two major subsets: pDCs and cDCs. Murine lymphoid-resident cDC subsets can be further divided into CD8α^+^ DCs and CD8α^−^ DCs (19, 20). Likewise, two distinct migratory cDC subsets are subcategorized based on CD103 and CD11b expressions in non-lymphoid tissues: CD103^+^CD11b^−^ cDCs and CD103^−^CD11b^+^ cDCs (21, 22). In humans, pDCs express TLR1, TLR7, and TLR9, while other DC subsets express all other TLRs except for TLR9 (23). In mice, both pDCs and cDCs express TLR2, TLR4, TLR7, and TLR9. In response to signaling through TLR2, TLR3, and TLR4, hepatic cDCs produce TNFα and IL-6 (24). However, recent reports showed that murine cDCs can produce an anti-inflammatory cytokine, IL-10, through TLR9 following ischemia/reperfusion injury (25). Hepatic pDCs produce inflammatory cytokines in response to TLR7 and TLR9 (24, 26, 27). Of note, a new subset of CCR9^+^ pDCs was identified as tolerogenic pDCs in an acute graft-versus-host disease model (28). Our group demonstrated CCR9^+^ pDCs exist abundantly within the murine liver, produce IL-10, and transforming growth factor β (TGFβ) and differentiate naïve T cells to a regulatory phenotype through TLR7 and TLR9 signaling (29).

### Lymphocytes

Intrahepatic lymphocytes account for about 25% of the non-parenchymal cells in the liver. They consist of NK, NKT, γδ T, αβ T, and B cells. Hepatic NK cells express TLRs1, 2, 3, 4, 6, 7, 8, and 9 and respond to the corresponding TLR ligands (30). TLR3 ligands negatively regulate liver regeneration via activation of NK cells (31). In general, T cells are indirectly activated by TLR signaling, but direct activation of T cells by TLR signaling through TLR2, 3, and 9 has been reported (32, 33).

### Hepatic stellate cells

Hepatic stellate cells account for a very small proportion (<1%) of non-parenchymal cells in the liver. Following liver injury, activated HSCs produce extracellular matrix components in the liver, such as collagen types 1, 3, and 4, leading to liver fibrosis (34). Activated human HSCs express TLR4 and CD14, and respond to LPS with the secretion of proinflammatory cytokines (35). Activated mouse HSCs express TLR2, TLR4, and TLR9, and respond to the corresponding ligands with the secretion of IL-6, vascular cell adhesion molecule 1 (VCAM-1), TGFβ1, and monocyte chemoattractant protein-1 (MCP-1) (36–38).

### Liver sinusoidal endothelial cells

Liver sinusoidal endothelial cells account for about 50% of non-parenchymal cells in the liver. LSECs express mRNAs for TLR1–9 and respond to the corresponding ligands except for that of TLR5. LSECs respond to TLR1, 2, 4, 6, 9 ligands by producing TNFα, and respond to TLR3 ligands by producing TNFα, IL-6, and interferon (IFN) (27). After repetitive LPS challenge, sinusoidal endothelial cells reduce NF-κB activation and mediate liver tolerance to maintain hepatic homeostasis (39). In the same way, LSECs play a role in maintaining the homeostasis of the liver through induction of antigen-specific T cell tolerance (40). A recent report demonstrated that LSECs mediate angiogenesis and subsequent liver fibrosis via TLR4 signaling (41).

### Hepatocytes

Primary cultured hepatocytes express TLR1–9, but only respond to TLR2 and TLR4 ligands (42). In the steady state, the responses to TLR2 and TLR4 are weak, while the expression of TLR2 and responsiveness to ligands is enhanced under inflammatory conditions (43, 44). Of note, hepatocytes, in concert with TLR4, CD14, and MD-2 play a role in the uptake and removal of LPS from the systemic circulation (45–47).

## Role of TLRs in Murine and Human Liver Injury

### Experimental acute liver injury (Concanavalin A)

**Figure 2:**
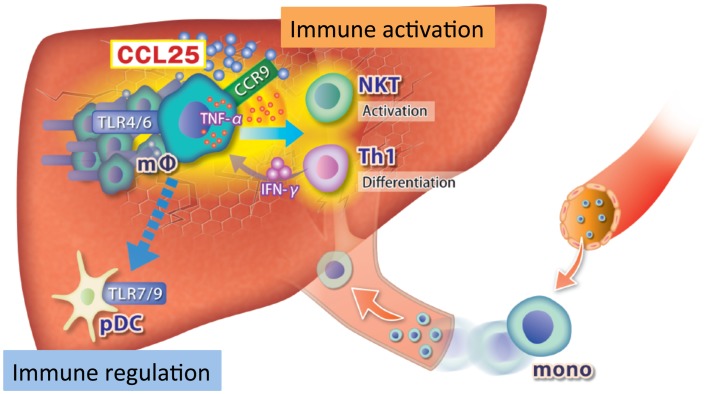
**Role of innate immune cells in the pathogenesis of Con A-induced acute liver injury**. Following Con A administration, CCL25 expression is up-regulated in the inflamed liver and CCR9^+^ macrophages accumulate to this site, while pDCs are down-regulated. CCR9^+^ macrophages produce TNFα and promote proliferation of IFNγ-producing Th1 and NKT cells via TLR4/6.

A single intravenous injection of Con A triggers acute liver injury in mice. It is accepted that Con A-induced acute liver injury is mediated mainly by CD3^+^CD4^+^NK1.1^+^NKT cells and CD3^+^CD4^+^NK1.1^-^ T cells (48–50). However, liver antigen-presenting cells (APCs) including KCs and DCs might be critically involved in the pathogenesis of Con A-induced liver injury, since it is significantly suppressed in KC-depleted mice (51–53). Signaling through TLR2, TLR3, TLR4, and TLR9 has been reported to contribute to liver injury in this model, especially in the early phase (54–56). We recently reported that TNFα-producing CCR9^+^CD11b^+^CD11c^−^macrophages expressed TLR2, TLR4, and TLR6 mRNAs and had a major role in the pathogenesis of acute liver injury in this model by activating Th1 and NKT cells (25). Of note, in the inflamed liver the number of tolerogenic CCR9^+^CD11b^−^CD11c^+^ pDCs that express TLR7 and TLR9 mRNAs decreases following Con A injection, suggesting the balance between inflammation and tolerance might be regulated by distinct immune cell subsets and TLRs in this model (Figure 2). Following Con A injection, up-regulation of TLR3 expression is observed in liver mononuclear cells and LSECs. The pathological role of TLR3 in this model was confirmed as TLR3^−/−^ mice were protected from Con A-induced hepatitis (57). In contrast, it was reported that Poly-I:C pretreatment activated NK cells and subsequently protected against Con A-mediated liver injury via down-regulation of T/NKT cells (58). Importantly, the protective effect of TLR3 was also reported in an LPS/D-GaiN-induced acute liver injury model (14). These results collectively indicate that TLR3 signaling has pleiotropic functions and is involved in inflammation, regeneration, and tolerance during the course of acute liver injury. The contribution of TLR9 in this model is controversial. TLR9 activation by CpG oligodeoxynucleotides (CpG-ODN) can exacerbate Con A-induced liver injury by promoting the activation of hepatic CD4^+^ NKT cells. The effect of TLR9 signaling on hepatic NKT cells was dependent on KCs and IL-12 (59). However, another report showed that pretreatment with CpG-ODN protected mice from Con A-induced hepatic injury by attenuating the activation of inflammatory cells (60). These contradictory findings could have resulted from differences in the DNA sequences used, because a different DNA sequences might trigger TLR9 signaling with different consequences, such as the release of potentially harmful (TNFα) or beneficial (IL-12) cytokines (61). Immunological tolerance to Con A was demonstrated as repeated Con A injection within 8 days after an initial Con A injection significantly reduced hepatic injury (62). The authors of that study concluded that CD4^+^CD25^+^ Tregs, KCs, and IL-10 were required for Con A tolerance. Further studies are required to clarify the contribution of specific TLRs and their downstream signaling to Con A tolerance.

### Ischemia–reperfusion and liver transplantation

Ischemia–reperfusion (I/R) injury is most commonly seen in the early period after liver transplantation. Recipients transplanted with livers from TLR4-deficient mice exhibited less I/R injury than those transplanted with wild-type livers (63), suggesting the inflammatory response seen in I/R injury is mainly mediated by TLR4. An alternative explanation is that TLR4 plays an indirect role by exacerbating I/R, as opposed to initiating the pathology. TLR4 expression on non-parenchymal cells is up-regulated by damage-associated molecular pattern molecules (DAMPs), such as high-mobility group box 1 protein (HMGB1) released from damaged hepatocytes during I/R (64, 65). Regarding allograft rejection and tolerance in liver transplantation, hepatic TLR4 expression has a distinctive role in CD8 T cell apoptosis and memory T cell generation (66). Increased TLR4-mediated expression of adhesion molecules in LSECs and KCs following continuous LPS exposure promoted trapping of T cells within the liver, resulting in lower numbers of circulating primed CD8 T cells and weak immune responses (39). The balance between alloimmune responsiveness and tolerance might be mediated by the level of TLR ligands that act as PAMPs or DAMPs, in association with clinical events such as I/R injury and infection after transplantation (67). It was recently reported that IL-10-producing cDCs reduced liver I/R injury in mice via TLR9 (25). Although the liver can mount an appropriate and sometimes excessive immune response to eliminate invading organisms, the overall balance appears to favor a state of immune permissiveness. As critical regulators of both innate and adaptive immunity, hepatic cDCs might play a role in orchestrating immune responses to limit undesirable inflammation and promote tolerance via TLR9. It is still unclear how the immune system can distinguish between threats from pathogens and endogenous danger signals, and contribute to both immune activation and tolerance through TLR9 signaling.

### Hepatitis B and Hepatitis C viral infection

The gene expression of TLR1, TLR2, TLR4, TLR6, TLR7, and TLR9 was decreased in peripheral blood mononuclear cells (PBMCs) from chronic Hepatitis B virus (HBV) infected patients, compared with healthy controls (68, 69). Impaired cytokine production with TLR2 and TLR4 ligands was also observed in PBMCs from chronic Hepatitis B (CHB) patients (68). Several TLR signaling pathways induce antiviral effects by up-regulating IFNs. Activation of TLR3, TLR4, TLR5, TLR7, and TLR9 by ligands mediates the inhibition of viral replication in HBV transgenic mice (70, 71). Importantly, HBV infection also induces immunosuppressive effects through TLR signaling. Overexpression of TLR2 and TLR4 on monocytes is reported to account for persistent HBV infection by modulating Treg functions (72). In PBMCs from chronic Hepatitis C virus (HCV) infected patients, the gene expression of TLR2, TLR3, TLR4, TLR6, TLR7 was increased (73, 74). HCV activates innate immune receptors including TLRs and retinoic acid-inducible gene 1 (RIG-I) to induce a chronic inflammatory state. Concurrently, HCV suppresses specific intracellular signaling to evade the host immune control (75). HCV core and NS3 proteins trigger TLR1, TLR2, and TLR6 on monocytes to enhance the production of inflammatory cytokines (76, 77). However, NS3/4a proteins degrade TIR-domain-containing adapter-inducing IFN-β (TRIF) and inhibit TLR3-mediated TRIF-dependent IFN-β production (78, 79). Furthermore, NS5 inhibits the recruitment of IL-1 receptor-associated kinase 1 (IRAK1), resulting in a decrease in TLRs-Myd88-dependent signals (80). An appropriate T cell response is required to eradicate HBV and HCV, while exhausted HCV-specific T cells with inhibitory immune receptors, such as PD-1 and CTLA-4, account for persistent viral infection within the liver (3, 4, 81, 82). LSECs with up-regulated PD-L1 expression were reported to induce antigen-specific T cell tolerance (40), and recent reports indicated that stimulation of LSECs with TLR1/2 ligands, but not TLR3 or TLR4 ligands could overcome liver-specific tolerance (83). Further study is required to clarify the effect of TLR1/2 ligands on the function of tolerant HBV- and HCV-specific T cells.

### Alcohol-induced liver disease

Excessive alcohol intake induces elevated levels of LPS in the liver through the portal circulation (84). The mechanism involved in the elevation of LPS is thought to be as follows. First, ingested alcohol disrupts the intestinal mucosal barrier and causes enhanced permeability (85, 86). Second, alcohol consumption leads to changes in the intestinal flora (87), and they migrate to liver sinusoids through the portal vein. KCs are a major target of LPS in various liver injuries including alcohol-induced liver injury (35, 88), as demonstrated by reduced liver inflammation following KC depletion (89). Recent reports indicated that TLR4 signaling in alcoholic liver injury was mediated through a MyD88-independent, but TRIF-dependent pathway (90, 91).

### Non-alcoholic steatohepatitis

Accumulating evidence indicates that LPS/TLR4 is also involved in the development of non-alcoholic steatohepatitis (NASH). A role for LPS in NASH was demonstrated by the finding that genetically obese *ob/ob* mice were sensitive to low-dose LPS (92). Furthermore, when fed a methionine/choline-deficient (MCD) diet, the most widely accepted experiment model of NASH, TLR4-deficient mice exhibited less severe hepatic injury and less accumulation of intrahepatic lipids compared with wild-type mice (93). These findings indicated activated TLR4 signaling pathways were critically involved in the pathogenesis of NASH. Recently, up-regulation of CD14 in KCs and hypersensitivity against low-dose LPS were observed in mice with high-fat diet (HFD)-induced steatosis (94). Hypersensitivity against low-dose LPS leads to accelerated NASH progression, including liver inflammation and fibrosis. In contrast, TLR2-deficient mice were not protected from steatohepatitis induced by MCD diet, affirming the TLR4 dependence of disease progression in this model (95). Notably, probiotics relieve the severity of NASH in leptin-deficient *ob/ob* mice, suggesting alterations of the intestinal flora might affect proinflammatory responses by disease-specific immune components through TLRs (96, 97).

### Liver fibrosis

Studies demonstrated elevated plasma LPS levels in experimental liver fibrosis induced by carbon tetrachloride (CCl_4_), thioacetamide, and bile duct ligation (BDL). TLR4 is expressed on both parenchymal and non-parenchymal cells in the liver, and several animal studies support the contribution of TLR4 in the development of liver fibrosis (36, 98, 99). Mice deficient for TLR4, CD14, MyD88, or TRIF exhibit reduced liver fibrosis in experimental fibrosis models (36, 98). In a recent study, Seki et al., clearly demonstrated that TLR4 on HSCs, but not on KCs or hepatocytes, was crucial for inducing liver fibrosis (36). Low concentrations of LPS can activate HSCs via TLR4 and downstream signaling to secrete a number of chemokines and adhesion molecules. These chemokines not only induce the migration of macrophages into the liver but also directly activate HSCs, leading to liver fibrosis. The role of chemokine receptors CCR1, CCR2, CCR5, CCR8, and CCR9 in liver fibrosis has been reported (100–104). A human study analyzing a large patient cohort demonstrated that certain single nucleotide polymorphisms (SNPs) in TLR4 were associated with reduced risk of liver cirrhosis in patients with chronic hepatitis C (105). The participation of TLR9 during liver fibrosis has been demonstrated in several mouse models of liver fibrosis, such as CCl_4_ and BDL models, in which TLR9-deficient mice exhibited significant reductions in liver fibrosis (106). Endogenous DNA from damaged hepatocytes is reported to enhance HSC activation through TLR9, thereby promoting liver fibrosis (37). TLR3 participates in the early stages of liver fibrosis but not during advanced liver fibrosis. Treatment with the TLR3 ligand Poly-I:C enhanced the activation of NK cells for killing HSCs, leading to attenuation of liver fibrosis (107). Recently, impaired TLR3 and TLR7/8 function was reported to affect rapid fibrosis progression post-liver transplantation with HCV infection (108).

### TLRs and microbiota

The translocation of intestinal microbiota into the liver and their recognition by TLRs results in both immune activation and tolerance under specific conditions. Importantly, this process is also critically involved in the development of a variety of liver diseases (109–112). Thus, targeting components of innate immune signaling, such as intestinal microbiota and TLRs may be an effective therapeutic approach to chronic liver diseases including viral hepatitis, alcoholic liver disease, NASH, and subsequent liver fibrosis. In particular, the mechanism of how endogenous TLR ligands associated with bacterial translocation contributes to immune activation and regulation, and subsequent chronic liver disease, should be comprehensively studied. Recent advances in gnotobiotic technology have enabled analysis of the role of specific bacterial strains in immunological responses (113–116). Using these techniques, a recent study reported that a complex mixture of 46 strains of *Clostridium* induced TGFβ in intestinal epithelial cells, which promoted the subsequent accumulation of IL-10-producing induced T regulatory cells, which in turn suppressed colitis in a dextran sodium sulfate colitis model (117). Very recently, our group reported that a single strain of *Clostridium butyricum* induced intestinal IL-10-producing macrophages via TLR2 and suppressed a mouse model of acute experimental colitis (118). Furthermore, butyrate-producing probiotics reduced the severity of murine NASH (119). These results clearly indicate that a single strain of microbiota can trigger immune activation and regulation via signaling through distinct TLRs. Further research should address in detail the crosstalk between disease-specific microbiota and the innate and adaptive immune system that occurs via specific TLRs signaling pathways in chronic liver diseases.

## Conclusion and Perspectives

The liver is continuously exposed to food antigens and PAMPs from the gastrointestinal tract via the portal vein. TLR signaling has a critical role in maintaining a balance between immune activation and tolerance. Following exposure to foreign antigens, TLRs are immediately activated and promote the induction of inflammatory cytokines and antimicrobial peptides to remove foreign microorganisms from the host. Concurrently, overactivation of TLRs that causes fetal events such as sepsis and acute liver failure should be controlled, which in turn might result in persistent infections in the liver. As described in this review, the following mechanisms have substantial roles in organ-specific tolerance: (1) hyporesponsiveness of individual TLR signaling due to the continuous exposure to ligands as seen in LPS tolerance (TLR4 on macrophages and LSECs) (17, 39), (2) the induction of other TLR signaling by DAMPs and host DNAs released from injured host cells and subsequent immunosuppressive cytokine production as seen in liver I/R injuries (TLR9 on cDCs) (25), and (3) dysfunctional antigen presentation by PD-L1-expressing APCs and the subsequent antigen-specific T cell exhaustion that can be reversed by TLR1/2 ligand stimulation as seen in chronic viral infections (TLR1/2 on LSECs) (83). Further studies, especially in humans, are required to clarify the interaction of each ligand-TLR signaling pathway on individual immune cell subsets that causes both immune activation and tolerance depending on severity and phase of the injury, and which eventually results in liver diseases such as chronic hepatitis, liver cirrhosis, and liver cancer. Understanding the underlying mechanisms in this area can aid the development of new therapeutic strategies in the future.

## Conflict of Interest Statement

The authors declare that the research was conducted in the absence of any commercial or financial relationships that could be construed as a potential conflict of interest.

## References

[1] Crispe IN The liver as a lymphoid organ. Annu Rev Immunol (2009) 27:147–63 10.1146/annurev.immunol.021908.132629 19302037

[2] Dong Z Wei H Sun R Tian Z The roles of innate immune cells in liver injury and regeneration. Cell Mol Immunol (2007) 4:241–52 17764614

[3] Nakamoto N Cho H Shaked A Olthoff K Valiga ME Kaminski M Synergistic reversal of intrahepatic HCV-specific CD8 T cell exhaustion by combined PD-1/CTLA-4 blockade. PLoS Pathog (2009) 5:e1000313 10.1371/journal.ppat.1000313 19247441 PMC2642724

[4] Nakamoto N Kaplan DE Coleclough J Li Y Valiga ME Kaminski M Functional restoration of HCV-specific CD8 T cells by PD-1 blockade is defined by PD-1 expression and compartmentalization. Gastroenterology (2008) 134:1927–37 10.1053/j.gastro.2008.02.033 18549878 PMC2665722

[5] Thomson AW Knolle PA Antigen-presenting cell function in the tolerogenic liver environment. Nat Rev Immunol (2010) 10:753–66 10.1038/nri2858 20972472

[6] Pasare C Medzhitov R Toll-like receptors: linking innate and adaptive immunity. Adv Exp Med Biol (2005) 560:11–8 10.1007/0-387-24180-9_2 15932016

[7] Takeuchi O Akira S Pattern recognition receptors and inflammation. Cell (2010) 140:805–20 10.1016/j.cell.2010.01.022 20303872

[8] De Creus A Abe M Lau AH Hackstein H Raimondi G Thomson AW Low TLR4 expression by liver dendritic cells correlates with reduced capacity to activate allogeneic T cells in response to endotoxin. J Immunol (2005) 174:2037–45 10.4049/jimmunol.174.4.2037 15699133

[9] Zarember KA Godowski PJ Tissue expression of human Toll-like receptors and differential regulation of Toll-like receptor mRNAs in leukocytes in response to microbes, their products, and cytokines. J Immunol (2002) 168:554–61 10.4049/jimmunol.168.2.554 11777946

[10] Tacke F Luedde T Trautwein C Inflammatory pathways in liver homeostasis and liver injury. Clin Rev Allergy Immunol (2009) 36:4–12 10.1007/s12016-008-8091-0 18600481

[11] Seki E Brenner DA Toll-like receptors and adaptor molecules in liver disease: update. Hepatology (2008) 48:322–35 10.1002/hep.22306 18506843

[12] Su GL Klein RD Aminlari A Zhang HY Steinstraesser L Alarcon WH Kupffer cell activation by lipopolysaccharide in rats: role for lipopolysaccharide binding protein and toll-like receptor 4. Hepatology (2000) 31:932–6 10.1053/he.2000.5634 10733550

[13] Seki E Tsutsui H Nakano H Tsuji N Hoshino K Adachi O Lipopolysaccharide-induced IL-18 secretion from murine Kupffer cells independently of myeloid differentiation factor 88 that is critically involved in induction of production of IL-12 and IL-1beta. J Immunol (2001) 166:2651–7 10.4049/jimmunol.166.4.2651 11160328

[14] Jiang W Sun R Wei H Tian Z Toll-like receptor 3 ligand attenuates LPS-induced liver injury by down-regulation of toll-like receptor 4 expression on macrophages. Proc Natl Acad Sci U S A (2005) 102:17077–82 10.1073/pnas.0504570102 16287979 PMC1287976

[15] Thobe BM Frink M Choudhry MA Schwacha MG Bland KI Chaudry IH Src family kinases regulate p38 MAPK-mediated IL-6 production in Kupffer cells following hypoxia. Am J Physiol Cell Physiol (2006) 291:C476–82 10.1152/ajpcell.00076.2006 16571868

[16] Kopydlowski KM Salkowski CA Cody MJ van Rooijen N Major J Hamilton TA Regulation of macrophage chemokine expression by lipopolysaccharide in vitro and in vivo. J Immunol (1999) 163:1537–44 10415057

[17] Knolle P Schlaak J Uhrig A Kempf P Meyer zum Büschenfelde KH Gerken G Human Kupffer cells secrete IL-10 in response to lipopolysaccharide (LPS) challenge. J Hepatol (1995) 22:226–9 10.1016/0168-8278(95)80433-1 7790711

[18] Duffield JS Forbes SJ Constandinou CM Clay S Partolina M Vuthoori S Selective depletion of macrophages reveals distinct, opposing roles during liver injury and repair. J Clin Invest (2005) 115:56–65 10.1172/JCI22675 15630444 PMC539199

[19] Vremec D Zorbas M Scollay R Saunders DJ Ardavin CF Wu L The surface phenotype of dendritic cells purified from mouse thymus and spleen: investigation of the CD8 expression by a subpopulation of dendritic cells. J Exp Med (1992) 176:47–58 10.1084/jem.176.1.47 1613465 PMC2119290

[20] Idoyaga J Steinman RM SnapShot: dendritic cells. Cell (2011) 146(660):e2 10.1016/j.cell.2011.08.010 21854989

[21] Geissmann F Manz MG Jung S Sieweke MH Merad M Ley K Development of monocytes, macrophages, and dendritic cells. Science (2010) 327:656–61 10.1126/science.1178331 20133564 PMC2887389

[22] Helft J Ginhoux F Bogunovic M Merad M Origin and functional heterogeneity of non-lymphoid tissue dendritic cells in mice. Immunol Rev (2010) 234:55–75 10.1111/j.0105-2896.2009.00885.x 20193012

[23] Edwards AD Diebold SS Slack EM Tomizawa H Hemmi H Kaisho T Toll-like receptor expression in murine DC subsets: lack of TLR7 expression by CD8 alpha+ DC correlates with unresponsiveness to imidazoquinolines. Eur J Immunol (2003) 33:827–33 10.1002/eji.200323797 12672047

[24] Shu SA Lian ZX Chuang YH Yang GX Moritoki Y Comstock SS The role of CD11c(+) hepatic dendritic cells in the induction of innate immune responses. Clin Exp Immunol (2007) 149:335–43 10.1111/j.1365-2249.2007.03419.x 17521321 PMC1941951

[25] Bamboat ZM Ocuin LM Balachandran VP Obaid H Plitas G DeMatteo RP Conventional DCs reduce liver ischemia/reperfusion injury in mice via IL-10 secretion. J Clin Invest (2010) 120:559–69 10.1172/JCI40008 20093775 PMC2810082

[26] Asselin-Paturel C Brizard G Chemin K Boonstra A O’Garra A Vicari A Type I interferon dependence of plasmacytoid dendritic cell activation and migration. J Exp Med (2005) 201:1157–67 10.1084/jem.20041930 15795237 PMC2213121

[27] Wu J Meng Z Jiang M Zhang E Trippler M Broering R Toll-like receptor-induced innate immune responses in non-parenchymal liver cells are cell type-specific. Immunology (2010) 129:363–74 10.1111/j.1365-2567.2009.03179.x 19922426 PMC2826681

[28] Hadeiba H Sato T Habtezion A Oderup C Pan J Butcher EC CCR9 expression defines tolerogenic plasmacytoid dendritic cells able to suppress acute graft-versus-host disease. Nat Immunol (2008) 9:1253–60 10.1038/ni.1658 18836452 PMC2901237

[29] Nakamoto N Ebinuma H Kanai T Chu PS Ono Y Mikami Y CCR9+ macrophages are required for acute liver inflammation in mouse models of hepatitis. Gastroenterology (2012) 142:366–76 10.1053/j.gastro.2011.10.039 22079594

[30] Sawaki J Tsutsui H Hayashi N Yasuda K Akira S Tanizawa T Type 1 cytokine/chemokine production by mouse NK cells following activation of their TLR/MyD88-mediated pathways. Int Immunol (2007) 19:311–20 10.1093/intimm/dxl148 17289654

[31] Sun R Gao B Negative regulation of liver regeneration by innate immunity (natural killer cells/interferon-gamma). Gastroenterology (2004) 127:1525–39 10.1053/j.gastro.2004.08.055 15521020

[32] Komai-Koma M Jones L Ogg GS Xu D Liew FY TLR2 is expressed on activated T cells as a costimulatory receptor. Proc Natl Acad Sci U S A (2004) 101:3029–34 10.1073/pnas.0400171101 14981245 PMC365739

[33] Gelman AE Zhang J Choi Y Turka LA Toll-like receptor ligands directly promote activated CD4+ T cell survival. J Immunol (2004) 172:6065–73 10.4049/jimmunol.172.10.6065 15128790 PMC2833313

[34] Bataller R Brenner DA Liver fibrosis. J Clin Invest (2005) 115:209–18 10.1172/JCI200524282 15690074 PMC546435

[35] Paik YH Schwabe RF Bataller R Russo MP Jobin C Brenner DA Toll-like receptor 4 mediates inflammatory signaling by bacterial lipopolysaccharide in human hepatic stellate cells. Hepatology (2003) 37:1043–55 10.1053/jhep.2003.50182 12717385

[36] Seki E De Minicis S Osterreicher CH Kluwe J Osawa Y Brenner DA TLR4 enhances TGF-beta signaling and hepatic fibrosis. Nat Med (2007) 13:1324–32 10.1038/nm1663 17952090

[37] Watanabe A Hashmi A Gomes DA Town T Badou A Flavell RA Apoptotic hepatocyte DNA inhibits hepatic stellate cell chemotaxis via toll-like receptor 9. Hepatology (2007) 46:1509–18 10.1002/hep.21867 17705260

[38] Guo J Loke J Zheng F Hong F Yea S Fukata M Functional linkage of cirrhosis-predictive single nucleotide polymorphisms of Toll-like receptor 4 to hepatic stellate cell responses. Hepatology (2009) 49:960–8 10.1002/hep.22697 19085953 PMC2891538

[39] Uhrig A Banafsche R Kremer M Hegenbarth S Hamann A Neurath M Development and functional consequences of LPS tolerance in sinusoidal endothelial cells of the liver. J Leukoc Biol (2005) 77:626–33 10.1189/jlb.0604332 15860798

[40] Limmer A Ohl J Kurts C Ljunggren HG Reiss Y Groettrup M Efficient presentation of exogenous antigen by liver endothelial cells to CD8+ T cells results in antigen-specific T-cell tolerance. Nat Med (2000) 6:1348–54 10.1038/82161 11100119

[41] Jagavelu K Routray C Shergill U O’Hara SP Faubion W Shah VH Endothelial cell toll-like receptor 4 regulates fibrosis-associated angiogenesis in the liver. Hepatology (2010) 52:590–601 10.1002/hep.23739 20564354 PMC2916032

[42] Liu S Gallo DJ Green AM Williams DL Gong X Shapiro RA Role of toll-like receptors in changes in gene expression and NF-kappa B activation in mouse hepatocytes stimulated with lipopolysaccharide. Infect Immun (2002) 70:3433–42 10.1128/IAI.70.7.3433-3442.2002 12065483 PMC128073

[43] Matsumura T Degawa T Takii T Hayashi H Okamoto T Inoue J TRAF6-NF-kappaB pathway is essential for interleukin-1-induced TLR2 expression and its functional response to TLR2 ligand in murine hepatocytes. Immunology (2003) 109:127–36 10.1046/j.1365-2567.2003.01627.x 12709026 PMC1782941

[44] Matsumura T Ito A Takii T Hayashi H Onozaki K Endotoxin and cytokine regulation of toll-like receptor (TLR) 2 and TLR4 gene expression in murine liver and hepatocytes. J Interferon Cytokine Res (2000) 20:915–21 10.1089/10799900050163299 11054280

[45] Fox ES Thomas P Broitman SA Clearance of gut-derived endotoxins by the liver. Release and modification of 3H, 14C-lipopolysaccharide by isolated rat Kupffer cells. Gastroenterology (1989) 96:456–61 2642878 10.1016/0016-5085(89)91571-0

[46] Mimura Y Sakisaka S Harada M Sata M Tanikawa K Role of hepatocytes in direct clearance of lipopolysaccharide in rats. Gastroenterology (1995) 109:1969–76 10.1016/0016-5085(95)90765-3 7498663

[47] Scott MJ Billiar TR Beta2-integrin-induced p38 MAPK activation is a key mediator in the CD14/TLR4/MD2-dependent uptake of lipopolysaccharide by hepatocytes. J Biol Chem (2008) 283:29433–46 10.1074/jbc.M803905200 18701460 PMC2570897

[48] Tiegs G Hentschel J Wendel A A T cell-dependent experimental liver injury in mice inducible by concanavalin A. J Clin Invest (1992) 90:196–203 10.1172/JCI115836 1634608 PMC443081

[49] Takeda K Hayakawa Y Van Kaer L Matsuda H Yagita H Okumura K Critical contribution of liver natural killer T cells to a murine model of hepatitis. Proc Natl Acad Sci U S A (2000) 97:5498–503 10.1073/pnas.040566697 10792025 PMC25857

[50] Kaneko Y Harada M Kawano T Yamashita M Shibata Y Gejyo F Augmentation of Valpha14 NKT cell-mediated cytotoxicity by interleukin 4 in an autocrine mechanism resulting in the development of concanavalin A-induced hepatitis. J Exp Med (2000) 191:105–14 10.1084/jem.191.1.105 10620609 PMC2195789

[51] Knolle PA Gerken G Loser E Dienes HP Gantner F Tiegs G Role of sinusoidal endothelial cells of the liver in concanavalin A-induced hepatic injury in mice. Hepatology (1996) 24:824–9 10.1002/hep.510240413 8855184

[52] Schümann J Wolf D Pahl A Brune K Papadopoulos T van Rooijen N Importance of Kupffer cells for T-cell-dependent liver injury in mice. Am J Pathol (2000) 157:1671–83 10.1016/S0002-9440(10)64804-3 11073826 PMC1885735

[53] Nakashima H Kinoshita M Nakashima M Habu Y Shono S Uchida T Superoxide produced by Kupffer cells is an essential effector in concanavalin A-induced hepatitis in mice. Hepatology (2008) 48:1979–88 10.1002/hep.22561 18942689

[54] Cao QY Chen F Li J Wu SS Wang J Chen Z A microarray analysis of early activated pathways in concanavalin A-induced hepatitis. J Zhejiang Univ Sci B (2010) 11:366–77 10.1631/jzus.B1000020 20443215 PMC2865839

[55] Ojiro K Ebinuma H Nakamoto N Wakabayashi K Mikami Y Ono Y MyD88-dependent pathway accelerates the liver damage of Concanavalin A-induced hepatitis. Biochem Biophys Res Commun (2010) 399:744–9 10.1016/j.bbrc.2010.08.012 20696131

[56] Sodhi A Tarang S Kesherwani V Concanavalin A induced expression of Toll-like receptors in murine peritoneal macrophages in vitro. Int Immunopharmacol (2007) 7:454–63 10.1016/j.intimp.2006.11.014 17321468

[57] Xiao X Zhao P Rodriguez-Pinto D Qi D Henegariu O Alexopoulou L Inflammatory regulation by TLR3 in acute hepatitis. J Immunol (2009) 183:3712–9 10.4049/jimmunol.0901221 19710451 PMC3787866

[58] Wang J Sun R Wei H Dong Z Gao B Tian Z Poly I:C prevents T cell-mediated hepatitis via an NK-dependent mechanism. J Hepatol (2006) 44:446–54 10.1016/j.jhep.2005.08.015 16310275

[59] Jiang W Sun R Zhou R Wei H Tian Z TLR-9 activation aggravates concanavalin A-induced hepatitis via promoting accumulation and activation of liver CD4+ NKT cells. J Immunol (2009) 182:3768–74 10.4049/jimmunol.0800973 19265155

[60] Zhang H Gong Q Li JH Kong XL Tian L Duan LH CpG ODN pretreatment attenuates concanavalin A-induced hepatitis in mice. Int Immunopharmacol (2010) 10:79–85 10.1016/j.intimp.2009.09.025 19818415

[61] Lipford GB Sparwasser T Bauer M Zimmermann S Koch ES Heeg K Immunostimulatory DNA: sequence-dependent production of potentially harmful or useful cytokines. Eur J Immunol (1997) 27:3420–6 10.1002/eji.1830271242 9464831

[62] Erhardt A Biburger M Papadopoulos T Tiegs G IL-10, regulatory T cells, and Kupffer cells mediate tolerance in concanavalin A-induced liver injury in mice. Hepatology (2007) 45:475–85 10.1002/hep.21498 17256743

[63] Shen XD Ke B Zhai Y Gao F Tsuchihashi S Lassman CR Absence of toll-like receptor 4 (TLR4) signaling in the donor organ reduces ischemia and reperfusion injury in a murine liver transplantation model. Liver Transpl (2007) 13:1435–43 10.1002/lt.21251 17902130

[64] Tsung A Sahai R Tanaka H Nakao A Fink MP Lotze MT The nuclear factor HMGB1 mediates hepatic injury after murine liver ischemia-reperfusion. J Exp Med (2005) 201:1135–43 10.1084/jem.20042614 15795240 PMC2213120

[65] Tsung A Hoffman RA Izuishi K Critchlow ND Nakao A Chan MH Hepatic ischemia/reperfusion injury involves functional TLR4 signaling in nonparenchymal cells. J Immunol (2005) 175:7661–8 10.4049/jimmunol.175.11.7661 16301676

[66] John B Klein I Crispe IN Immune role of hepatic TLR-4 revealed by orthotopic mouse liver transplantation. Hepatology (2007) 45:178–86 10.1002/hep.21446 17187407

[67] Howell J Gow P Angus P Visvanathan K Role of toll-like receptors in liver transplantation. Liver Transpl (2014) 20:270–80 10.1002/lt.23793 24243591

[68] Chen Z Cheng Y Xu Y Liao J Zhang X Hu Y Expression profiles and function of Toll-like receptors 2 and 4 in peripheral blood mononuclear cells of chronic hepatitis B patients. Clin Immunol (2008) 128:400–8 10.1016/j.clim.2008.04.006 18565796

[69] Xu N Yao HP Sun Z Chen Z Toll-like receptor 7 and 9 expression in peripheral blood mononuclear cells from patients with chronic hepatitis B and related hepatocellular carcinoma. Acta Pharmacol Sin (2008) 29:239–44 10.1111/j.1745-7254.2008.00711.x 18215354

[70] McClary H Koch R Chisari FV Guidotti LG Relative sensitivity of hepatitis B virus and other hepatotropic viruses to the antiviral effects of cytokines. J Virol (2000) 74:2255–64 10.1128/JVI.74.5.2255-2264.2000 10666256 PMC111707

[71] Isogawa M Robek MD Furuichi Y Chisari FV Toll-like receptor signaling inhibits hepatitis B virus replication in vivo. J Virol (2005) 79:7269–72 10.1128/JVI.79.11.7269-7272.2005 15890966 PMC1112123

[72] Zhang Y Lian JQ Huang CX Wang JP Wei X Nan XP Overexpression of Toll-like receptor 2/4 on monocytes modulates the activities of CD4(+)CD25(+) regulatory T cells in chronic hepatitis B virus infection. Virology (2010) 397:34–42 10.1016/j.virol.2009.11.007 19945134

[73] He Q Graham CS Durante Mangoni E Koziel MJ Differential expression of toll-like receptor mRNA in treatment non-responders and sustained virologic responders at baseline in patients with chronic hepatitis C. Liver Int (2006) 26:1100–10 10.1111/j.1478-3231.2006.01357.x 17032411

[74] Sato K Ishikawa T Okumura A Yamauchi T Sato S Ayada M Expression of Toll-like receptors in chronic hepatitis C virus infection. J Gastroenterol Hepatol (2007) 22:1627–32 10.1111/j.1440-1746.2006.04783.x 17845690

[75] Riordan SM Skinner NA Kurtovic J Locarnini S McIver CJ Williams R Toll-like receptor expression in chronic hepatitis C: correlation with pro-inflammatory cytokine levels and liver injury. Inflamm Res (2006) 55:279–85 10.1007/s00011-006-0082-0 16955390

[76] Dolganiuc A Oak S Kodys K Golenbock DT Finberg RW Kurt-Jones E Hepatitis C core and nonstructural 3 proteins trigger toll-like receptor 2-mediated pathways and inflammatory activation. Gastroenterology (2004) 127:1513–24 10.1053/j.gastro.2004.08.067 15521019

[77] Chang S Dolganiuc A Szabo G Toll-like receptors 1 and 6 are involved in TLR2-mediated macrophage activation by hepatitis C virus core and NS3 proteins. J Leukoc Biol (2007) 82:479–87 10.1189/jlb.0207128 17595379

[78] Li K Foy E Ferreon JC Nakamura M Ferreon AC Ikeda M Immune evasion by hepatitis C virus NS3/4A protease-mediated cleavage of the Toll-like receptor 3 adaptor protein TRIF. Proc Natl Acad Sci U S A (2005) 102:2992–7 10.1073/pnas.0408824102 15710891 PMC548795

[79] Otsuka M Kato N Moriyama M Taniguchi H Wang Y Dharel N Interaction between the HCV NS3 protein and the host TBK1 protein leads to inhibition of cellular antiviral responses. Hepatology (2005) 41:1004–12 10.1002/hep.20666 15841462

[80] Abe T Kaname Y Hamamoto I Tsuda Y Wen X Taguwa S Hepatitis C virus nonstructural protein 5A modulates the toll-like receptor-MyD88-dependent signaling pathway in macrophage cell lines. J Virol (2007) 81:8953–66 10.1128/JVI.00649-07 17567694 PMC1951400

[81] Radziewicz H Ibegbu CC Fernandez ML Workowski KA Obideen K Wehbi M Liver-infiltrating lymphocytes in chronic human hepatitis C virus infection display an exhausted phenotype with high levels of PD-1 and low levels of CD127 expression. J Virol (2007) 81:2545–53 10.1128/JVI.02021-06 17182670 PMC1865979

[82] Penna A Pilli M Zerbini A Orlandini A Mezzadri S Sacchelli L Dysfunction and functional restoration of HCV-specific CD8 responses in chronic hepatitis C virus infection. Hepatology (2007) 45:588–601 10.1002/hep.21541 17326153

[83] Liu J Jiang M Ma Z Dietze KK Zelinskyy G Yang D TLR1/2 ligand-stimulated mouse liver endothelial cells secrete IL-12 and trigger CD8+ T cell immunity in vitro. J Immunol (2013) 191:6178–90 10.4049/jimmunol.1301262 24227786

[84] Wheeler MD Endotoxin and Kupffer cell activation in alcoholic liver disease. Alcohol Res Health (2003) 27:300–6 15540801 PMC6668869

[85] Draper LR Gyure LA Hall JG Robertson D Effect of alcohol on the integrity of the intestinal epithelium. Gut (1983) 24:399–404 10.1136/gut.24.5.399 6840613 PMC1419986

[86] Bjarnason I Peters TJ Wise RJ The leaky gut of alcoholism: possible route of entry for toxic compounds. Lancet (1984) 1:179–82 10.1016/S0140-6736(84)92109-3 6141332

[87] Bode JC Bode C Heidelbach R Dürr HK Martini GA Jejunal microflora in patients with chronic alcohol abuse. Hepatogastroenterology (1984) 31:30–4 6698486

[88] Mandrekar P Szabo G Signalling pathways in alcohol-induced liver inflammation. J Hepatol (2009) 50:1258–66 10.1016/j.jhep.2009.03.007 19398236 PMC3342816

[89] Ganey PE Schultze AE Depletion of neutrophils and modulation of Kupffer cell function in allyl alcohol-induced hepatotoxicity. Toxicology (1995) 99:99–106 10.1016/0300-483X(94)03005-M 7762005

[90] Hritz I Mandrekar P Velayudham A Catalano D Dolganiuc A Kodys K The critical role of toll-like receptor (TLR) 4 in alcoholic liver disease is independent of the common TLR adapter MyD88. Hepatology (2008) 48:1224–31 10.1002/hep.22470 18792393 PMC7137387

[91] Petrasek J Dolganiuc A Csak T Nath B Hritz I Kodys K Interferon regulatory factor 3 and type I interferons are protective in alcoholic liver injury in mice by way of crosstalk of parenchymal and myeloid cells. Hepatology (2011) 53:649–60 10.1002/hep.24059 21274885 PMC3069538

[92] Yang SQ Lin HZ Lane MD Clemens M Diehl AM Obesity increases sensitivity to endotoxin liver injury: implications for the pathogenesis of steatohepatitis. Proc Natl Acad Sci U S A (1997) 94:2557–62 10.1073/pnas.94.6.2557 9122234 PMC20127

[93] Rivera CA Adegboyega P van Rooijen N Tagalicud A Allman M Wallace M Toll-like receptor-4 signaling and Kupffer cells play pivotal roles in the pathogenesis of non-alcoholic steatohepatitis. J Hepatol (2007) 47:571–9 10.1016/j.jhep.2007.04.019 17644211 PMC2094119

[94] Imajo K Fujita K Yoneda M Nozaki Y Ogawa Y Shinohara Y Hyperresponsivity to low-dose endotoxin during progression to nonalcoholic steatohepatitis is regulated by leptin-mediated signaling. Cell Metab (2012) 16:44–54 10.1016/j.cmet.2012.05.012 22768838

[95] Szabo G Velayudham A Romics L Jr Mandrekar P Modulation of non-alcoholic steatohepatitis by pattern recognition receptors in mice: the role of toll-like receptors 2 and 4. Alcohol Clin Exp Res (2005) 29:140S–5S 10.1097/01.alc.0000189287.83544.33 16344599

[96] Li Z Yang S Lin H Huang J Watkins PA Moser AB Probiotics and antibodies to TNF inhibit inflammatory activity and improve nonalcoholic fatty liver disease. Hepatology (2003) 37:343–50 10.1053/jhep.2003.50048 12540784

[97] Solga SF Diehl AM Non-alcoholic fatty liver disease: lumen-liver interactions and possible role for probiotics. J Hepatol (2003) 38:681–7 10.1016/S0168-8278(03)00097-7 12713883

[98] Isayama F Hines IN Kremer M Milton RJ Byrd CL Perry AW LPS signaling enhances hepatic fibrogenesis caused by experimental cholestasis in mice. Am J Physiol Gastrointest Liver Physiol (2006) 290:G1318–28 10.1152/ajpgi.00405.2005 16439470

[99] Wu JB Chuang HR Yang LC Lin WC A standardized aqueous extract of Anoectochilus formosanus ameliorated thioacetamide-induced liver fibrosis in mice: the role of Kupffer cells. Biosci Biotechnol Biochem (2010) 74:781–7 10.1271/bbb.90824 20378990

[100] Chu PS Nakamoto N Ebinuma H Usui S Saeki K Matsumoto A C-C motif chemokine receptor 9 positive macrophages activate hepatic stellate cells and promote liver fibrosis in mice. Hepatology (2013) 58:337–50 10.1002/hep.26351 23460364

[101] Seki E De Minicis S Gwak GY Kluwe J Inokuchi S Bursill CA CCR1 and CCR5 promote hepatic fibrosis in mice. J Clin Invest (2009) 119:1858–70 10.1172/JCI37444 19603542 PMC2701864

[102] Seki E de Minicis S Inokuchi S Taura K Miyai K van Rooijen N CCR2 promotes hepatic fibrosis in mice. Hepatology (2009) 50:185–97 10.1002/hep.22952 19441102 PMC2705470

[103] Karlmark KR Weiskirchen R Zimmermann HW Gassler N Ginhoux F Weber C Hepatic recruitment of the inflammatory Gr1+ monocyte subset upon liver injury promotes hepatic fibrosis. Hepatology (2009) 50:261–74 10.1002/hep.22950 19554540

[104] Heymann F Hammerich L Storch D Bartneck M Huss S Rüsseler V Hepatic macrophage migration and differentiation critical for liver fibrosis is mediated by the chemokine receptor C-C motif chemokine receptor 8 in mice. Hepatology (2012) 55:898–909 10.1002/hep.24764 22031018 PMC4533854

[105] Li Y Chang M Abar O Garcia V Rowland C Catanese J Multiple variants in toll-like receptor 4 gene modulate risk of liver fibrosis in Caucasians with chronic hepatitis C infection. J Hepatol (2009) 51:750–7 10.1016/j.jhep.2009.04.027 19586676 PMC2883297

[106] Gäbele E Mühlbauer M Dorn C Weiss TS Froh M Schnabl B Role of TLR9 in hepatic stellate cells and experimental liver fibrosis. Biochem Biophys Res Commun (2008) 376:271–6 10.1016/j.bbrc.2008.08.096 18760996

[107] Jeong WI Park O Radaeva S Gao B STAT1 inhibits liver fibrosis in mice by inhibiting stellate cell proliferation and stimulating NK cell cytotoxicity. Hepatology (2006) 44:1441–51 10.1002/hep.21419 17133483

[108] Howell J Sawhney R Skinner N Gow P Angus P Ratnam D Toll-like receptor 3 and 7/8 function is impaired in hepatitis C rapid fibrosis progression post-liver transplantation. Am J Transplant (2013) 13:943–53 10.1111/ajt.12165 23425350

[109] Mencin A Kluwe J Schwabe RF Toll-like receptors as targets in chronic liver diseases. Gut (2009) 58:704–20 10.1136/gut.2008.156307 19359436 PMC2791673

[110] Hartmann P Chen WC Schnabl B The intestinal microbiome and the leaky gut as therapeutic targets in alcoholic liver disease. Front Physiol (2012) 3:402 10.3389/fphys.2012.00402 23087650 PMC3468817

[111] Seki E Schnabl B Role of innate immunity and the microbiota in liver fibrosis: crosstalk between the liver and gut. J Physiol (2012) 590:447–58 10.1113/jphysiol.2011.219691 22124143 PMC3379693

[112] Darnaud M Faivre J Moniaux N Targeting gut flora to prevent progression of hepatocellular carcinoma. J Hepatol (2013) 58:385–7 10.1016/j.jhep.2012.08.019 22940407

[113] Josefowicz SZ Niec RE Kim HY Treuting P Chinen T Zheng Y Extrathymically generated regulatory T cells control mucosal TH2 inflammation. Nature (2012) 482:395–9 10.1038/nature10772 22318520 PMC3485072

[114] Chinen T Rudensky AY The effects of commensal microbiota on immune cell subsets and inflammatory responses. Immunol Rev (2012) 245:45–55 10.1111/j.1600-065X.2011.01083.x 22168413

[115] Geuking MB Cahenzli J Lawson MA Ng DC Slack E Hapfelmeier S Intestinal bacterial colonization induces mutualistic regulatory T cell responses. Immunity (2011) 34:794–806 10.1016/j.immuni.2011.03.021 21596591

[116] Tlaskalová-Hogenová H Stepánková R Kozáková H Hudcovic T Vannucci L Tucková L The role of gut microbiota (commensal bacteria) and the mucosal barrier in the pathogenesis of inflammatory and autoimmune diseases and cancer: contribution of germ-free and gnotobiotic animal models of human diseases. Cell Mol Immunol (2011) 8:110–20 10.1038/cmi.2010.67 21278760 PMC4003137

[117] Atarashi K Tanoue T Shima T Imaoka A Kuwahara T Momose Y Induction of colonic regulatory T cells by indigenous *Clostridium* species. Science (2011) 331:337–41 10.1126/science.1198469 21205640 PMC3969237

[118] Hayashi A Sato T Kamada N Mikami Y Matsuoka K Hisamatsu T A single strain of *Clostridium butyricum* induces intestinal IL-10-producing macrophages to suppress acute experimental colitis in mice. Cell Host Microbe (2013) 13:711–22 10.1016/j.chom.2013.05.013 23768495

[119] Endo H Niioka M Kobayashi N Tanaka M Watanabe T Butyrate-producing probiotics reduce nonalcoholic fatty liver disease progression in rats: new insight into the probiotics for the gut-liver axis. PLoS One (2013) 8:e63388 10.1371/journal.pone.0063388 23696823 PMC3656030

